# Loosening of Plates

**Published:** 1860-07

**Authors:** 


### ARTICLE III.

### *Loosening of Plates.*

ONE of the most singular attendants of a newly inserted plate, is that in almost all cases they become loose in about from ten to twenty days after their insertion and remain so for a short period, perhaps one week or ten days, when they again assume a permanent and useful state.

I am inclined to believe that this peculiar change occurs only in the most perfectly adapted plates, not having noticed it whilst using swaged plates, but only since my adoption of the Cheoplastic system, which gives an invariable correct fit, and finding that in all pieces, this period of partial non-adhesion always takes place at about the same time, lasting for the same length of time with a slight variation.

For a season I could hardly understand the cause which produced so sudden and so great a change. Plates would fit with the greatest perfection, and give immediate results, such results as can be realized only in this excellent mode, when in a few weeks patients would return, declaring that they were not so tight or firm as at first, equally comfortable, independent of a sense of their great liability to fall, asking the reason, which at first I found difficult to give, but latterly have at least satisfied myself upon this point, and offer my explanation to others, hoping to receive further enlightenment upon this subject from your journal.

A newly inserted plate, even in a mouth accustomed to wearing teeth, rests either upon an entire or partially new surface, always leaving some portion of the palate surface untouched. For a season the portion in contact, supports the plate satisfactorily, until from the irritation produced from the mechanical friction and pressure, the parts which have not touched the plate, swell and displace those parts which have been in contact and have supported the plate.

It may frequently happen that from the close approximation of the plate and palate surface, that at this time there may indeed be a perfect adaptation, only the mucous surface is soft and yielding, and from the irritation produced, must be followed by a condition of partial congestion of the fluid of the part, resulting in a free discharge of mucous secretion, of a sufficiently abnornal state, to be viscid and glazy, and the discharge of which must take place between the adhering surfaces, causing displacement and the introduction of air, of course attended by a loosening and increased insecurity of the plate.

At first, there must be in all plates, even in those made after this process, a nonperfect adaptation. The greater or less inaccuracy of impressions, many imperceptible changes resulting from however carefully conducted manipulations, from minute changes which doubtless takes place in the mouth, in the varying condition of health of the body affecting this part, temperature, activity of circulation, &c., &c., all producing change of volume in the mouth, giving variation at times of importance, from the condition when the impression has been taken and when the plate inserted.

The most perfect method would, by this, appear as only a comparative perfection, greatly dependent upon the skill, care or genius of the operator, *all plates requiring* more or less time to become absolutely accurate in the fit, and perfect in their usefulness. The subsidence of the mucous secretion takes place as the irritation becomes subdued by it, and is immediately followed by an induration of the membrane, with a general thickening, at the same time that all prominences are being absorbed and depressions filled up, until a very close approximation to exactitude of the two surfaces will be found. Use subsequently induces irregular absorption principally around the edge of plate, compensated for by a greater solidity of bearing upon the plate surface ; doubtless there are more or less changes going on in the mouth, through life, of the character of waste and repair.

With this process, I never hesitate to promise my patients the entire supply of their demands, but always explain the probable changes which will go on at first. So much confidence have I in it, that I boldly state that all pieces of dental mechanism can be made through it of the most perfect character and with entirely satisfactory results in the end. Not being disposed to write an encomium of the inventor or the process, I in justice state that I have continued to use it exclusively, and shall do so until something better shall be introduced. I am well satisfied it has not a rival at this time for true merit.

JAMES.

